# Facial minimally invasive aesthetic procedures and quality of life in transgender individuals: a prospective study^☆^

**DOI:** 10.1016/j.abd.2026.501316

**Published:** 2026-03-27

**Authors:** Walter Refkalefsky Loureiro, Francisca Regina Oliveira Carneiro, Letícia Rezende da Silva Sobral

**Affiliations:** a Service of Dermatology, Universidade do Estado do Pará, Belém, PA, Brazil; b Private Practice, Imperatriz, MA, Brazil

Dear Editor,

Transgender individuals, whose gender identity differs from the sex assigned at birth, increasingly seek aesthetic procedures to align their appearance with their self-identified gender. Among the available tools, injectable fillers and neuromodulators play an important role in facial gender affirmation, offering a safe and minimally invasive alternative to surgery. However, little is known about their impact on Quality of Life (QoL). This study evaluates changes in QoL following facial injectable procedures in transgender patients.

We conducted a prospective, interventional study at the Dermatology Service of the Pará State University, Belém (PA), Brazil, enrolling 20 transgender adults ‒ 10 trans women and 10 trans men ‒ aged 22- to 47-years (mean ± SD, 30.5 ± 7.3). All participants had been on continuous hormone therapy for at least two years and had no prior facial aesthetic procedures. Informed consent was obtained, and the protocol was approved by the local ethics committee (CAAE 58177222.6.0000.5174). All individuals were injected by the same dermatologist, including touch-ups if needed. Patients were recruited at a public center that oversees hormonal treatment for transgender individuals. The authors are affiliated with a public dermatology institution, but injections were performed in the private practice of one author.

Procedures were personalized to enhance gender-specific traits, based on established aesthetic patterns and patient preferences. Trans women were treated to create arched eyebrows, fuller lips, malar projection, and a rounded chin. Trans men underwent procedures to strengthen the jawline, project the chin, and straighten the eyebrows. AbobotulinumtoxinA (Dysport®, Ipsen, Wrexham – UK) and hyaluronic acid fillers (Restylane® range, Galderma, Uppsala – Sweden) were used, and all treatments were performed in a single session, with neuromodulator touch-ups when necessary. Treatment protocols are detailed in Fig. 1A.

**Fig. 1:**
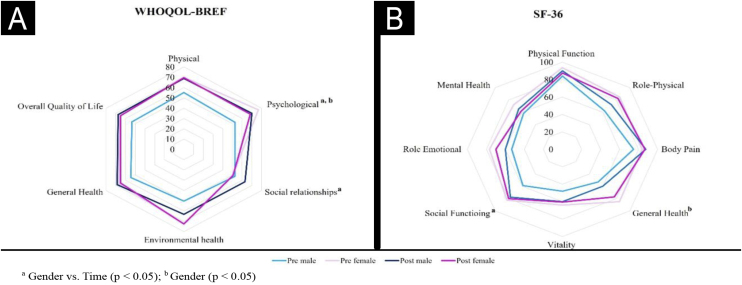
Radar chart presenting each domain scores for WHOQOL (A) and SF-26 (B) questionnaires, considering the scores at baseline and after 30-days of treatment by gender. The comparative analysis showed significant differences when comparing gender vs. time in psychological and social relationship domains of WHOQOL, and the social functioning domain of SF-36. In addition, both psychological (WHOQOL) and general health (SF-36) domains presented significant differences in the gender comparison alone.

QoL was assessed using two validated instruments: WHOQOL-BREF and SF-36, applied at baseline and 30-days post-treatment. WHOQOL-BREF evaluates four domains ‒ physical health, psychological, social relationships, and environmental ‒ and includes two global items on general health and overall QoL. SF-36 encompasses eight components, including physical functioning, social functioning, and mental health. The WHO-QOL BREF assesses well-being over two weeks, while the SF-36 covers four weeks. This 30-days’ time return balances recollection bias and unrelated changes, avoids exceeding recommended toxin injection intervals and reduces dropouts.

Post-treatment results showed statistically significant improvements in WHOQOL-BREF domains of physical health (p = 0.01), psychological well-being (p = 0.048), and overall QoL (p = 0.03). These effects were particularly marked among trans men, who showed significant gains in psychological (p = 0.02) and social relationship domains (p = 0.003) (Fig. 1B). While the SF-36 scores did not show significant differences for the cohort as a whole, trans men reported higher post-treatment scores in social functioning (p = 0.042).

The results highlight the potential of injectable procedures to enhance the well-being of transgender individuals by reinforcing gender-congruent facial traits and promoting self-perception and social integration. This aligns with previous evidence that gender-affirming interventions can mitigate psychosocial stress and improve quality of life in this population.^1^, ^2^, ^3^, ^4^

Interestingly, while trans women also reported improvements, the effects were more modest. This may be related to social determinants, hormonal response variability, or the higher aesthetic expectations often placed on feminizing procedures. Moreover, injectable treatments alone may not be sufficient to achieve the degree of facial transformation desired by some trans women, particularly those with more masculine skeletal structures.^5^

This study supports the use of minimally invasive facial procedures as a valuable tool for gender affirmation, especially for patients not yet ready or eligible for surgical interventions. The procedures were well tolerated, and no adverse events were reported during the follow-up. The personalized, gender-informed approach used in this study may serve as a practical reference for clinicians working with transgender patients.

Limitations include the small sample size, short follow-up, and absence of a control group. Furthermore, broader psychosocial factors such as discrimination, social support, and employment status were not controlled for but may influence QoL independently of aesthetic changes. Nevertheless, these preliminary findings provide encouraging data on the benefits of aesthetic dermatology in this context and warrant further investigation in larger and longer-term studies. A noteworthy example of a public initiative in transgender care in Argentina was recently published in ABD, highlighting the management of clinical conditions both related and unrelated to hormone therapy, such as acne, androgenic alopecia, and siliconomas.^6^ A publicly funded ambulatory service for transgender patients is being developed at the University to offer free care. Funding negotiations for injectables with the local government are ongoing.

## ORCID ID

Francisca Regina Oliveira Carneiro: 0000-0001-6735-4004

Letícia Rezende da Silva Sobral: 0009-0002-6724-1549

## Financial support

None declared.

## Authors' contributions

Walter Refkalefsky Loureiro: Approval of the final version of the manuscript; critical literature review; data collection, analysis and interpretation; effective participation in research orientation; intellectual participation in propaedeutic and/or therapeutic management of studied cases; manuscript critical review; preparation and writing of the manuscript; statistical analysis; study conception and planning.

Francisca Regina Oliveira Carneiro: Approval of the final version of the manuscript; effective participation in research orientation; manuscript critical review.

Letícia Rezende da Silva Sobral: Critical literature review; intellectual participation in propaedeutic and/or therapeutic management of studied cases; data collection, analysis and interpretation.

## Research data availability

The entire dataset supporting the results of this study was published in this article.

## Conflicts of interest

Dr. Walter R. Loureiro serves as a speaker for Galderma. However, no financial compensation or support was received for the conduct of this study.
